# Current trends and geographical differences in therapeutic profile and outcomes of COVID-19 among pregnant women - a systematic review and meta-analysis

**DOI:** 10.1186/s12884-021-03685-w

**Published:** 2021-03-24

**Authors:** Pallavi Dubey, Bhaskar Thakur, Sireesha Reddy, Carla A. Martinez, Md Nurunnabi, Sharron L. Manuel, Sadhana Chheda, Christina Bracamontes, Alok K. Dwivedi

**Affiliations:** 1 Department of Obstetrics and Gynecology, Paul L. Foster School of Medicine, Texas Tech University Health Sciences Center El Paso, El Paso, TX USA; 2 Division of Biostatistics & Epidemiology, Department of Molecular and Translational Medicine, Paul L. Foster School of Medicine, Texas Tech University Health Sciences Center El Paso, El Paso, TX USA; 3 School of Pharmacy, the University of Texas at El Paso, El Paso, TX USA; 4 Department of Pediatrics, Paul L. Foster School of Medicine, Texas Tech University Health Sciences Center El Paso, El Paso, TX USA; 5 Biostatistics and Epidemiology Consulting Lab, Texas Tech University Health Sciences Center El Paso, El Paso, TX USA

**Keywords:** Treatment, Hydroxychloroquine, Antivirals, Preterm birth, Antibiotics, Mechanical ventilation, ICU admission, Maternal death, Adverse pregnancy outcomes, Meta-analysis, Cesarean section

## Abstract

**Background:**

Coronavirus disease (COVID-19) has been associated with adverse pregnancy outcomes. Due to the lack of effective treatments for COVID-19, it becomes imperative to assess the geographical differences and trends in the current clinical care and outcomes of COVID-19 in pregnant women.

**Methods:**

A PubMed search was performed to screen articles reporting therapeutics and outcomes of confirmed COVID-19 in pregnant women prior to August 27, 2020. We performed searches, quality assessments of eligible studies, extracted and reported data according to PRISMA guidelines. Meta-analyses and cumulative meta-analyses of proportions were performed for estimating each outcome and their pattern over time respectively.

**Results:**

One thousand two hundred thirty nine pregnant women with COVID-19 from 66 studies were analyzed. In case series analysis reflecting average-risk patients, the proportion of oxygen support, antibiotics, antivirals, and plasma therapy administration except for hydroxychloroquine was substantially higher in Asian studies (55, 78, 80, 6, and 0%) compared to the US (7, 1, 12, 0, and 7%) or European (33, 12, 14, 1, and 26%) studies, respectively. The highest preterm birth and the average length of hospital stay (35%, 11.9 days) were estimated in Asian studies compared to the US studies (13%, 9.4 days) and European studies (29%, 7.3 days), respectively. Even in case reports reflecting severe cases, the use of antivirals and antibiotics was higher in Asian studies compared to the US, Latin American, and European studies. A significant decline in the use of most therapeutics along with adverse outcomes of COVID-19 in pregnant women was observed.

**Conclusions:**

Geographical differences in therapeutic practice of COVID-19 were observed with differential rates of maternal and clinical outcomes. Minimizing the use of some therapeutics particularly antibiotics, antivirals, oxygen therapy, immunosuppressants, and hydroxychloroquine by risk stratification and careful consideration may further improve maternal and clinical outcomes.

**Supplementary Information:**

The online version contains supplementary material available at 10.1186/s12884-021-03685-w.

## Background

An estimated 27 million people worldwide have been infected with the coronavirus disease 2019 till October 2020 [1, 2]. SARS-CoV-2 infection seems less virulent than the Severe Acute Respiratory Syndrome (SARS) and Middle East Respiratory Syndrome (MERS) in terms of morbidity and mortality [3, 4]. We and others have observed high rates of adverse pregnancy outcomes including preterm birth among COVID-19 women [5]. There are no established therapies for COVID-19 particularly in pregnant women. Hence, it becomes imperative to provide observational evidence of the current therapeutic practice of COVID-19 in clinical care for the management of pregnant women.

The most common therapeutics for managing COVID-19 in pregnant women were antibiotics, antivirals, and oxygen supports. Intensive care and mechanical ventilation (MV) is needed to deal with disease severity [6]. Lopinavir /Ritonavir, a HIV-1 protease inhibitor [7] has been used as a treatment option for COVID-19 as an antiviral, however, there is no clear benefit observed in the treatment of COVID-19 [8]. In contrast, remdesivir [9] and dexamethasone [10] are considered acceptable treatments with evidence for hospitalized and severe COVID-19 patients. Tocilizumab as an IL-6 inhibitor has been used for treating severe and critical COVID-19 cases in the US [11]. Although hydroxychloroquine (HCQ) initially received emergency use authorization and then revoked by the Food and Drug Administration [12], HCQ has been used for treating COVID-19 patients [13]. The use of antibiotics has also been observed in COVID-19 cases without any bacterial infection [14]. Convalescent plasma therapy and anticoagulants have been recommended for the treatment of hospitalized COVID-19 patients and critical patients [15, 16]. In addition, the potential benefits of zinc/magnesium have been demonstrated especially in elderly or immunocompromised patients and thus these treatments have been used for managing COVID-19 as well [17].

Information on maternal and pregnancy outcomes after treatment for COVID-19 has been limited [18]. During ongoing COVID-19 pandemic, understanding the chronological pattern of therapeutic use as per their benefits and risks corresponding to the pattern in maternal and clinical outcomes may help health care management to make an appropriate decision in the increase or decrease use of specific therapy or medication. In our previous study [19], we observed the geographical differences in pregnancy outcomes showing more prevalent adverse pregnancy outcomes in China compared to the US and Europe. Although pregnant women have an increased risk of hospitalization and intensive care unit (ICU) admission [6], it is still unknown if there are any geographical differences persist in COVID-19 outcomes as well.

## Methods

### Search methods

**Fig. 1:**
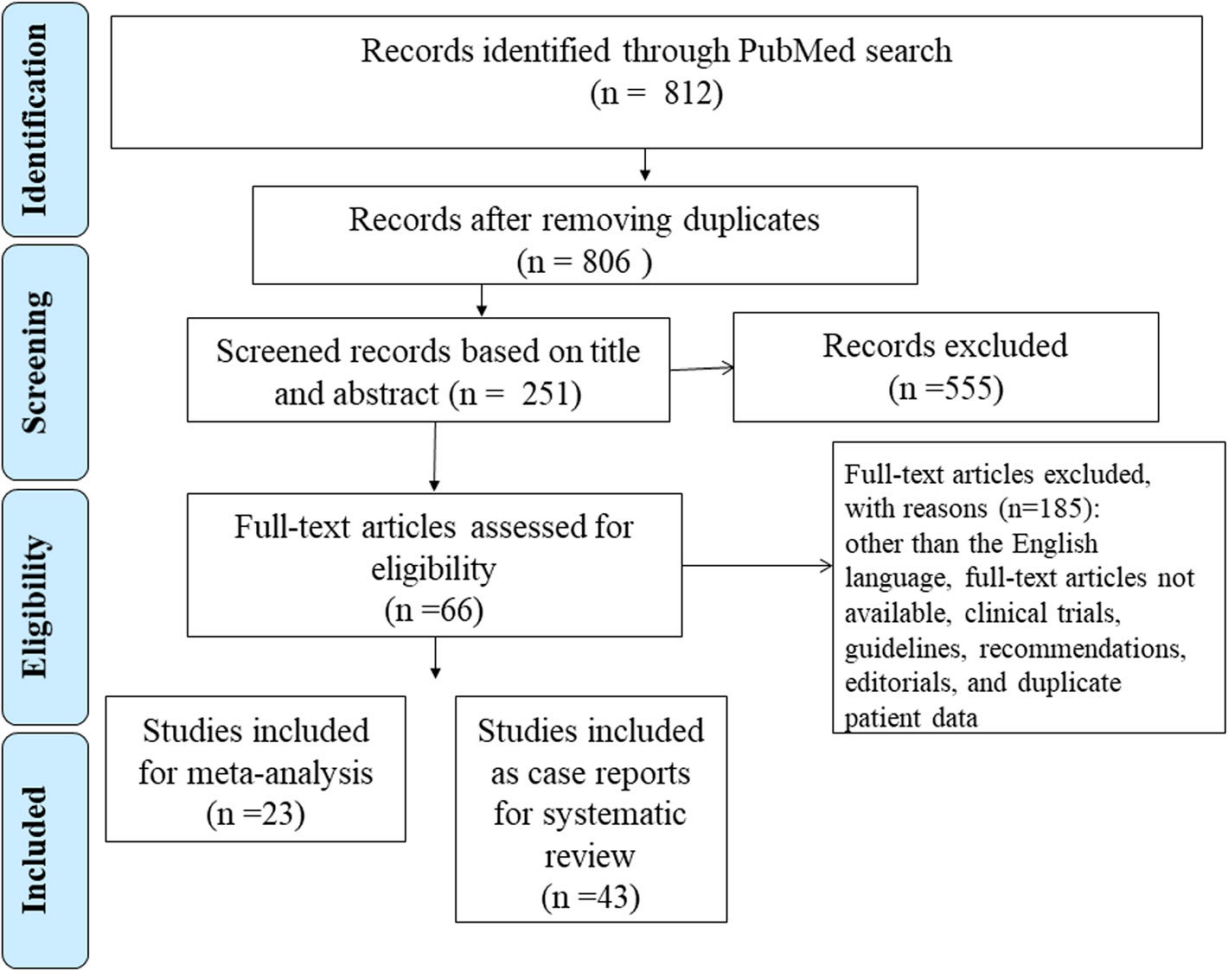
PRISMA flowchart for study selection

We followed the Preferred Reporting Items for Systematic Reviews and Meta-analyses (PRISMA) [20] and the Meta-analysis of Observational Studies in Epidemiology (MOOSE) [21] guidelines [22]. A comprehensive search was performed on PubMed for screening any studies reporting data on COVID-19 in pregnant women prior to August 27th, 2020. We used the combinations of search terms “COVID-19 OR SARS-CoV-2 OR Coronavirus” AND “Pregnancy OR Pregnant” to screen articles. References and review studies were utilized to screen any eligible studies excluded from the initial search (Fig. 1). The two authors (PD and SR) independently reviewed all studies for their eligibility. An extensive review of all articles was conducted by two authors (BT and PD) to exclude any duplicated articles based on the recruitment period, location, or authors of studies.

### Inclusion and exclusion criteria

Any study reporting treatments for managing SARS-CoV-2 infection in pregnant women in outpatients setting or during hospitalization was considered eligible for this systematic review and meta-analysis study. All pregnant patients with a confirmed COVID-19 infection by quantitative real-time polymerase chain reaction with or without maternal or clinical outcomes were only included in this study. Unpublished studies/reports and studies that could not be translated into the English language or indicating duplicative data were excluded from the analysis.

### Study endpoints

We extracted data on a) treatment profile including oxygen support, corticosteroids, immunosuppressants, HCQ, antivirals, zinc/magnesium, anticoagulants, antibiotics, plasma therapy, and mechanical ventilation b) maternal and neonatal outcomes including pregancy status (delivered or still pregnant), number of neonates, delivery type (cesarean section or vaginal), maternal death, fetal demise, neonatal death, c) clinical outcomes of COVID-19 including intensive care unit (ICU) admission and hospital length of stay (HLOS) d) maternal characteristics including age range, gestational age, comorbidities (diabetes-DM, hypertension-HTN, asthma, obesity), symptomatic or asymptomatic status of COVID-19. The primary outcomes in the study were therapeutics for managing COVID-19 in pregnant women, preterm birth, ICU admission, and HLOS. Maternal death and fetal demise rates were included as secondary outcomes. Studies with more than 5 cases were considered as case series studies otherwise case reports. The location of the study was classified into Asia, United States of America (USA), Europe, and Latin America. However, data from Latin American countries was only available in case reports studies.

### Assessments of the risk of bias

We conducted the quality assessment of all eligible case series studies using the quality assessment tool provided by the National Heart, Lung, and Blood Institute, Research Triangle Institute International. In addition, we performed an assessment of publication bias using funnel plots and Egger’s test for primary outcomes only.

### Statistical analysis

For case series studies, the random effects meta-analysis of proportions was carried out for each binary outcome using the Dersimonian and Laird (D-L) method with the Freeman-Tukey double arcsine transformation to obtain the estimates. The results of the D-L method were summarized using a pooled proportion with a 95% confidence interval (CI). The I^2^ statistic was used to assess the heterogeneity across effect sizes. The average HLOS was pooled across the studies using the weighted generalized linear model (GLM) with the Gaussian family and identity link function. The studies were weighted according to the sample size of each study, larger sample size studies received a higher weight relative to smaller sample size studies. Cumulative meta-analysis for each outcome was performed by the month of each publication to obtain a pattern in outcome over the pandemic period. The subgroup analyses for ICU, HLOS, and preterm birth were also conducted using the D-L random effects models or weighted GLM according to low or high use of each of the therapeutics, comorbidities, cesarean section rate, and symptomatic clinical presentation. For the systematic review of case reports, we performed individual level descriptive data analysis and applied a Fisher’s exact test or an unpaired t-test or a Pearson’s correlation analysis. All the statistical computations were carried out by the STATA statistical software (version 15.1).

## Results

Eight hundred twelve studies were screened and two hundred fifty-one were evaluated for data extraction. Of 251 studies, 66 fulfilled the eligibility criteria yielding 1239 COVID-19 cases for data analysis. Twenty-three studies were included in meta-analysis while 43 studies reporting 64 cases were included in the systematic review and qualified for descriptive data analysis (Table 1). Qualitative assessment and bias evaluation were performed on all the 23 studies eligible for the meta-analysis. Most of the studies included in the meta-analysis were of fair/good quality (Supplementary Table 1) without any sign of publication bias (Supplementary Table 2, Supplementary Fig. 1 A and B).

**Table 1:** Study characteristics

PMID	Author	Month	Country	N	Age(y)	GW	BMI (kg/m^**2**^)	DM	HT	Asthma	Obesity	ICU	HLOS (days)	Preterm
**Case series**														
32151335	Chen et al. [23]	March	China	9	33	37.1	NA	NA	NA	NA	NA	0	NA	4
32186894	Liu et al. [24]	March	China	15	31.5	25	NA	1	0	0	0	NA	NA	3
32249918	Li et al. [25]	March	China	16	31.5	36.5	NA	3	3	0	0	0	14.5	13
32285380	Liu et al. [26]	March	China	19	31	38	NA	NA	NA	NA	NA	0	NA	2
32360108	Hantoushzadeh et al. [27]	April	Iran	9	37	43	27	1	0	0	1	9	NA	6
32428964	London et al. [28]	April	USA	68	29.6	NA	30.25	7	2	2		1	14.5	9
32696241	Chen et al. [29]	April	China	21	29	NA	NA	3	0	0	0	0		
32433453	Savasi et al. [30]	May	Italy	77	31.5	23	35.8	NA	NA	NA	NA	14	NA	12
32438521	Zeng et al. [31]	May	China	16	32.5	37	NA	NA	NA	NA	NA	0	NA	3
32439389	Lokken et al. [32]	May	USA	46	30	27.4	NA	3	2	4	15	1	4.5	1
32632417	San-Juan et al. [33]	May	Spain	32	32	29	NA	2	0	4	1	2	7.5	NA
32641013	Zhang et al. [34]	May	China	18	29	38	NA	NA	NA	NA	NA	0	NA	3
32553908	Sentilhes et al. [35]	June	France	54	30.6	30.4	25.3	0	1	5	4	4	4	NA
32553910	Blitz et al. [36]	June	USA	43	29.5	35	30.9	1	0	2	5	13	16	1
32633022	Prabhu et al. [37]	July	USA	70	31.6	38.6	31	6	11	6	12	1	3.6	11
32633712	Vivanti et al. [38]	July	France	100	32.8	27	27.05	7	6	9	10	10	9.1	20
32649784	Gabriel et al. [39]	July	Spain	42	33.6	37.6	NA	NA	NA	NA	NA	3	NA	9
32682342	Sahin et al. [40]	July	Turkey	29	28.5	22	28	0	0	1	5	0	11	2
32689846	Barbero et al. [41]	July	Spain	91	33.25	28	26.2	3	3	5	20	4	NA	8
32701761	Emeruwa et al. [42]	July	USA	100	32	28	30.7	3	17	12	NA	NA	NA	13
32743014	Xu et al. [43]	July	China	34	32.5	28	NA	2	1	0	0	1	10.6	5
32776309	Oncel et al. [44]	August	Turkey	125	NA	34.5	NA	7	8	0	NA	8	6.5	33
32760169	Nayak et al. [45]	August	India	141	25	35.5	NA	4	7	2	NA	NA	NA	NA
**Summary**				**1175**	**30.60**	**31.30**	**29.40**	**53**	**61**	**52**	**73**	**71**	**8.47**	**158**
**Case report**														
32119083	Wang et al. [46]	February	China	1	28	30	NA	NA	NA	NA	NA	0	19	1
32134381	Li et al. [47]	March	China	1	30	35	NA	0	0	0	0	0	15	1
32161941	Wang et al. [48]	March	China	1	34	40	NA	0	0	0	0	0	18	0
32182347	Fan et al. [49]	March	China	2	31.5	36.2	NA	NA	NA	NA	NA	0	20	1
32222119	Chen et al. [50]	March	China	5	28	39.5	NA	NA	NA	NA	NA	0	NA	0
32229802	Lee et al. [51]	March	Korea	1	35	37	NA	NA	NA	NA	NA	1	1	0
32249471	Kalafat et al. [52]	March	Turkey	1	32	35	NA	0	0	0	0	1	9	1
32249924	Gidlöf et al. [53]	March	Sweden	1	34	36	38	1	0	0	1	0	NA	2
32279693	Khan et al. [54]	March	China	3	30	36.5	NA	NA	NA	NA	NA	0	NA	1
32237670	Iqbal et al. [55]	April	USA	1	34	39	NA	NA	NA	NA	NA	0	6	0
32305046	Alzamora et al. [56]	April	Peru	1	41	33	35	0	0	0	2	0	3	1
32305459	Peng et al. [57]	April	China	1	25	35	NA	NA	NA	NA	NA	0	14	1
32313302	Romero et al. [58]	April	Spain	1	44	29	NA	0	0	0	0	1	NA	NA
32330313	Lu et al. [59]	April	China	1	22	38	NA	0	0	0	0	0	16	0
32330970	Browne et al. [60]	April	USA	1	33	23	NA	0	0	1	0	0	4	0
32384385	Blauvelt et al. [61]	April	USA	1	34	28	NA	1	0	1	1	1	16	1
32509416	Silverstein et al. [62]	April	USA	2	25.5	35	40	0	0	0	1	2	19	2
32523874	AlZagha et al. [63]	April	Jordan	1	30	36	NA	0	0	0	0	0	11	1
32369616	Li et al. [64]	May	China	1	31	35	NA	0	0	0	0	1	45	1
32382516	Hong et al. [65]	May	USA	1	36	23	41.53	0	0	0	1	1	11	0
32405454	Taghizadieh et al. [66]	May	Iran	1	33	34	NA	0	0	0	0	1	NA	1
32425297	Cooke et al. [67]	May	UK	2	33.5	28	42	1	0	0	1	0	7	2
32426242	Mehta et al. [68]	May	USA	1	39	27	NA	0	0	0	0	1	11	2
32426243	Anderson et al. [8]	May	USA	1	35	22	NA	1	0	1	1	0	14	0
32428290	Yu et al. [69]	May	China	1	35	34	NA	0	0	0	0	1	12	1
32505514	Fontanella et al. [70]	May		2	34	35.5	43	1	0	0	0	0	3.5	0
32606133	Grimminck et al. [71]	June	Netherlands	1	31	38	NA	0	1	0	0	0	1	0
32618794	Naqvi et al. [72]	June	USA	1	35	22	28	1	1	1	0	1	9	0
32702930	Zheng et al. [73]	June	China	2	31	37.5	NA	0	0	0	0	2	28.5	1
32716009	Douedi et al. [74]	June	USA	3	23	29.5	NA	NA	NA	NA	NA	3	9	3
32740456	Marzollo et al. [75]	June	Italy	1	29	38	NA	0	0	0	0	1	18	0
32667391	Reis et al. [76]	July	Brazil	3	29.5	34	NA	0	0	0	0	3	18	2
32675129	Oliva et al. [77]	July	USA	1	35	29	NA	1	0	0	NA	1	15	1
32704477	Richtmann et al. [78]	July	Brazil	5	32	25.5	27.7	0	0	0	2	0	NA	0
32714844	Kolkova et al. [79]	July	Sweden	1	27	32	57	1	0	0	1	1	31	1
32715804	Soleimani et al. [80]	July	Iran	1	30	21	36	0	0	0	1	1	31	0
32723092	Easterlin et al. [81]	July	USA	1	22	23	NA	NA	NA	NA	NA	1	28	1
32754425	Chong et al. [82]	July	USA	1	41	32	35.6	0	0	0	1	1	12	1
32784239	Ahmed et al. [83]	July	UK	1	26	37	25	1	1	0	0	0	7	0
32773854	Chhabra et al. [84]	May	India	1	28	38	31.5	1	0	0	0	0	22	0
32784234	Figueiredo et al. [85]	July	Portugal	1	35	39	NA	0	0	0	0	0	15	0
32788159	Federici et al. [86]	August	France	1	33	23.5	NA	0	1	0	NA	1	19	1
32791731	Peng et al. [87]	June	China	1	33	38	NA	NA	NA	NA	NA	0	5	0
**Summary**				**64**	**31.10**	**32.72**	**35.80**	**10** **(23.3)**	**4** **(9.3)**	**4** **(9.3)**	**12** **(29.3)**	**27 (43.5)**	**14.6** **(9.0)**	**32** **(50.8)**

*PMID *PubMed identifier*, N* number of subjects, *GW* gestational age measured in weeks, *BMI* body mass index, *DM* diabetes mellitus, *HT* hypertension, *ICU* intensive care unit, *HLOS* hospital length of stay, *NA* not available, *UK* United Kingdom*, USA* United States of America

### Maternal characteristics, treatment profile, and outcomes

In the analysis of the 1239 pregnant women, 944 subjects had a delivery while 295 women were still pregnant at the end of the study. In case series, the average maternal age was 30.6 years (mid-range: 25–37) with average gestational age of 31.3 (mid-range: 22–43) weeks. The average BMI was 29.4 (mid-range: 25.3–35.8) kg/m^2^. Overall 9% of patients had obesity and 4% had DM, HTN, or asthma. Most patients presented with a symptom of COVID-19 (89%). The most common therapeutic was antibiotics (36%) followed by oxygen support (33%), antivirals (33%), HCQ (10%), anticoagulants (3%), and plasma support (2%). The proportion of mechanical ventilation was estimated to be 3% with a 6% ICU admission rate (95%CI: 2, 10%). The average HLOS was 8.5 days (95%CI: 5.96, 10.97). The cesarean section and preterm birth rates were estimated to be 62 and 26% respectively. Fetal demise was less than 1% with 20 maternal deaths (Table 2).

**Table 2:** Overall estimates of maternal characteristics, treatment profile, and outcomes

	Case series	Case reports	***P***-value
	Proportion (95% CI)	Proportion (95% CI)	
**Maternal Characteristics**			
Age (years) mean (95% CI)	30.60 (28.90, 32.40)^a^	31.10 (29.97, 32.24)	0.359
BMI (Kg/m^2^) mean (95% CI)	29.40 (27.30, 31.70)^a^	35.81 (32.04, 39.57)	< 0.001
Gestational age (weeks) mean (95% CI)	31.30 (28.80, 33.80)^a^	32.70 (31.31, 34.24)	0.050
**Symptomatic presentation**	0.89 (0.75, 0.98)	0.89 (0.78, 0.95)	0.006
**Comorbidities**			
Diabetes mellitus	0.04 (0.03, 0.07)	0.23 (0.12, 0.39)	< 0.001
Hypertension	0.04 (0.02, 0.07)	0.09 (0.03, 0.22)	0.283
Asthma	0.04 (0.02, 0.06)	0.09 (0.03, 0.22)	0.131
Obesity	0.09 (0.04, 0.15)	0.29 (0.16, 0.46)	< 0.001
**Treatment**			
Oxygen support	0.33 (0.20, 0.47)	0.53 (0.40, 0.66)	< 0.001
Steroids	0.06 (0.00, 0.19)	0.44 (0.31, 0.57)	< 0.001
Immunosuppressants	0.01 (0.00, 0.03)	0.10 (0.04, 0.20)	0.004
Hydroxychloroquine	0.10 (0.03, 0.19)	0.26 (0.16, 0.38)	0.082
Antivirals	0.33 (0.18, 0.49)	0.45 (0.32, 0.58)	< 0.001
Zinc/Magnesium	0.02 (0.00, 0.14)	0.21 (0.12, 0.34)	0.007
Anticoagulants	0.03 (0.00, 0.07)	0.12 (0.05, 0.23)	0.031
Antibiotics	0.36 (0.21, 0.52)	0.64 (0.51, 0.76)	< 0.001
Plasma therapy/Anti-liver damage	0.02 (0.00, 0.06)	0.14 (0.07, 0.26)	< 0.001
Mechanical ventilation	0.03 (0.01, 0.07)	0.35 (0.23, 0.49)	< 0.001
**Maternal & Pregnancy Outcomes**			
ICU admission	0.06 (0.02, 0.10)	0.43 (0.31, 0.57)	< 0.001
Hospital length of stay (days) mean (95% CI)	8.47 (5.96, 10.97)^a^	14.6 (12.0, 17.2)	< 0.001
Maternal death	0.003 (0.00, 0.02)	0.05 (0.01, 0.13)	0.108
Cesarean section	0.62 (0.54, 0.71)	0.69 (0.56, 0.80)	0.085
Fetal demise	< 1%	0.08 (0.03, 0.19)	0.003
Neonatal death	< 1%	0.02 (0.00, 0.08)	0.143
Premature birth	0.26 (0.19, 0.34)	0.50 (0.37, 0.63)	< 0.001

^a^Weighted mean reported; *I*^*2*^ % measure of heterogeneity, *CI* confidence interval, *ICU* intensive care unit, *NA* not applicable, *BMI* body mass index

In case reports, the average maternal age, gestational age, and average BMI were 31.1 (mid-range: 22–44) years, 32.7 (mid-range: 21–40) weeks, and 35.8 (mid-range: 25–57) kg/m^2^ respectively. The most common comorbidities were obesity (30%) and DM (23%) followed by HTN and asthma (9%). The majority of patients had a symptomatic presentation (89%). The proportion of antibiotics, oxygen support, antivirals, steroids, HCQ, zinc/magnesium, plasma therapy, anticoagulants, and immunosuppressants was 64, 53, 45, 44, 26, 21, 14, 12, and 10% respectively. Over one-third of the patients required mechanical ventilation support (35%) with 43% ICU admission rate (95%CI: 31, 57%). The average HLOS was estimated to be 14.6 days (95%CI: 12.0–17.2). The rate of cesarean section, preterm birth, fetal demise, and maternal death was 69, 50, 8, and 5% respectively. In addition, 2% neonatal deaths were recorded (Table 2).

### Meta-analysis of maternal characteristics, treatment profile, and outcomes by geographic location

The average age of patients was highest in Europe (32.1 years) followed by the US (30.8 years) and Asia (28.4 years). However, the average gestational age at detection was lowest in European studies (29.3 weeks) and the US studies (31.9 weeks) compared to Asian studies (34.8 weeks). Most of the pregnant patients were symptomatic in Asian (100%) and European (92%) studies, while almost half of the patients were asymptomatic in the US studies. Among comorbidities, obesity was most commonly reported in the US (20%) and European studies (11%). Among therapeutics, antibiotics were most commonly observed in Asia (78%) while oxygen support (33%) and HCQ (26%) were the most common therapeutics in Europe. Although with low proportions, antibiotics (12%), HCQ (7%), and oxygen support (7%) were relatively more used in the US patients compared to other treatments. The least use of any therapeutics except for HCQ was obtained in the US studies relative to other countries. European studies had higher proportions of HCQ and corticosteroid use than the US and Asia. The proportions of mechanical ventilation support (2, 4, and 6%) and ICU admission (6, 5, and 7%) were found to be similar across the US, Asia, and Europe, respectively. However, the average HLOS was highest in Asian studies (11.8 days) and least in European studies (7.34 days). The preterm birth rate was relatively higher in Asian (35%) and European studies (29%) compared to the US studies (13%). The cesarean section rate was similar between US (46%) and European studies (53%) but lower than Asian studies (80%) (Table 3).

**Table 3:** Meta-analysis of maternal characteristics, therapeutics, and outcomes by geographic location

	Asia			USA			Europe		
	N	I^**2**^	Proportion (95% CI)	N	I^**2**^	Proportion (95% CI)	N	I^**2**^	Proportion (95% CI)
**Maternal Characteristics**									
Age (years); mean (95% CI)^a^	10	NA	28.40 (24.80, 32.10)	5	NA	30.80 (29.60, 31.90)	7	NA	30.60 (28.90, 32.40)
BMI (kg/m^2^); mean (95% CI)^a^	1	NA	27.00 (single study)	4	NA	30.60 (30.40, 31.00)	5	NA	29.40 (27.30, 31.70)
Gestational age (weeks); mean (95% CI)^a^	9	NA	34.80 (32.40, 37.20)	4	NA	31.90 (26.20, 37.70)	8	NA	31.30 (28.80, 33.80)
**Symptomatic Presentation**	6	0.0	1.00 (0.96, 1.00)	4	97.1	0.54 (0.21, 0.85)	8	93.4	0.92 (0.80, 0.99)
**Comorbidities**									
Diabetes mellitus	6	46.1	0.07 (0.02, 0.14)	5	26.0	0.06 (0.03, 0.09)	6	45.2	0.03 (0.01, 0.06)
Hypertension	6	16.5	0.03 (0.00, 0.07)	5	82.8	0.07 (0.01, 0.15)	6	21.8	0.03 (0.01, 0.06)
Asthma	6	0.0	0.00 (0.00, 0.01)	5	24.2	0.07 (0.04, 0.11)	6	79.6	0.05 (0.01, 0.11)
Obesity	5	0.0	0.00 (0.00, 0.03)	3	ID	0.20 (0.10, 0.32)	5	64.8	0.11 (0.06, 0.19)
**Treatment**									
Oxygen support	9	94.9	0.55 (0.20, 0.88)	5	59.0	0.07 (0.03, 0.12)	6	84.2	0.33 (0.21, 0.46)
Steroids	9	73.5	0.05 (0.00, 0.16)	4	83.8	0.01 (0.00, 0.09)	8	98.8	0.10 (0.00, 0.44)
Immunosuppressants	9	0.0	0.00 (0.00, 0.02)	4	87.9	0.02 (0.00, 0.11)	7	83.3	0.02 (0.00, 0.07)
Hydroxychloroquine	9	43.2	0.00 (0.00, 0.04)	5	84.0	0.07 (0.02, 0.17)	8	96.6	0.26 (0.09, 0.49)
Antivirals	9	89.8	0.80 (0.56, 0.97)	4	59.1	0.01 (0.00, 0.05)	8	89.8	0.12 (0.04, 0.22)
Zinc/Magnesium	9	78.5	0.02 (0.00, 0.11)	4	0.0	0.00 (0.00, 0.01)	8	99.0	0.05 (0.00, 0.39)
Anticoagulant	9	67.9	0.02 (0.00, 0.09)	4	92.2	0.03 (0.00, 0.17)	8	91.8	0.04 (0.00, 0.12)
Antibiotics	9	95.4	0.78 (0.41, 1.00)	5	84.3	0.12 (0.04, 0.22)	8	94.1	0.14 (0.04, 0.29)
Plasma therapy/Anti-liver damage	9	93.1	0.06 (0.00, 0.3)	5	20.3	0.00 (0.00, 0.01)	8	81.3	0.01 (0.00, 0.04)
Mechanical ventilation	9	86.8	0.04 (0.00, 0.18)	4	84.6	0.02 (0.00, 0.11)	8	0.0	0.06 (0.04, 0.08)
**Maternal & Pregnancy Outcomes**									
ICU admission	8	87.9	0.05 (0.00, 0.23)	4	88.8	0.06 (0.00, 0.19)	8	52.7	0.07 (0.04, 0.11)
HLOS (days) mean (95% CI)^a^	2	NA	11.86 (8.55, 15.17)	4	NA	9.40 (2.96, 15.83)	5	NA	7.34 (5.46, 9.23)
Maternal death	10	71.5	0.01 (0.00, 0.07)	5	20.3	0.00 (0.00, 0.01)	8	32.9	0.01 (0.00, 0.02)
Cesarean section	10	85.1	0.80 (0.61, 0.94)	5	0.0	0.46 (0.39, 0.53)	8	72.3	0.53 (0.41, 0.66)
Fetal demise	2	ID	0.02 (0.00, 0.05)	2	ID	0.01 (0, 0.04)	1	ID	0.02 (0.00, 0.10)
Premature birth	8	77.8	0.35 (0.17, 0.55)	5	0.0	0.13 (0.09, 0.18)	6	65.1	0.29 (0.20, 0.40)

Note: ^a^Weighted mean reported; Asia includes China, India and Iran; Europe includes France, Italy, Spain and Turkey

*I*^*2*^ % measure of heterogeneity, *CI* confidence interval, *ICU* intensive care unit, *HLOS* hospital length of stay, *ID* insufficient data, *NA* not applicable, *BMI* body mass index, *USA* United States of America

### Cumulative meta-analysis of treatments and outcomes over time

**Fig. 2:**
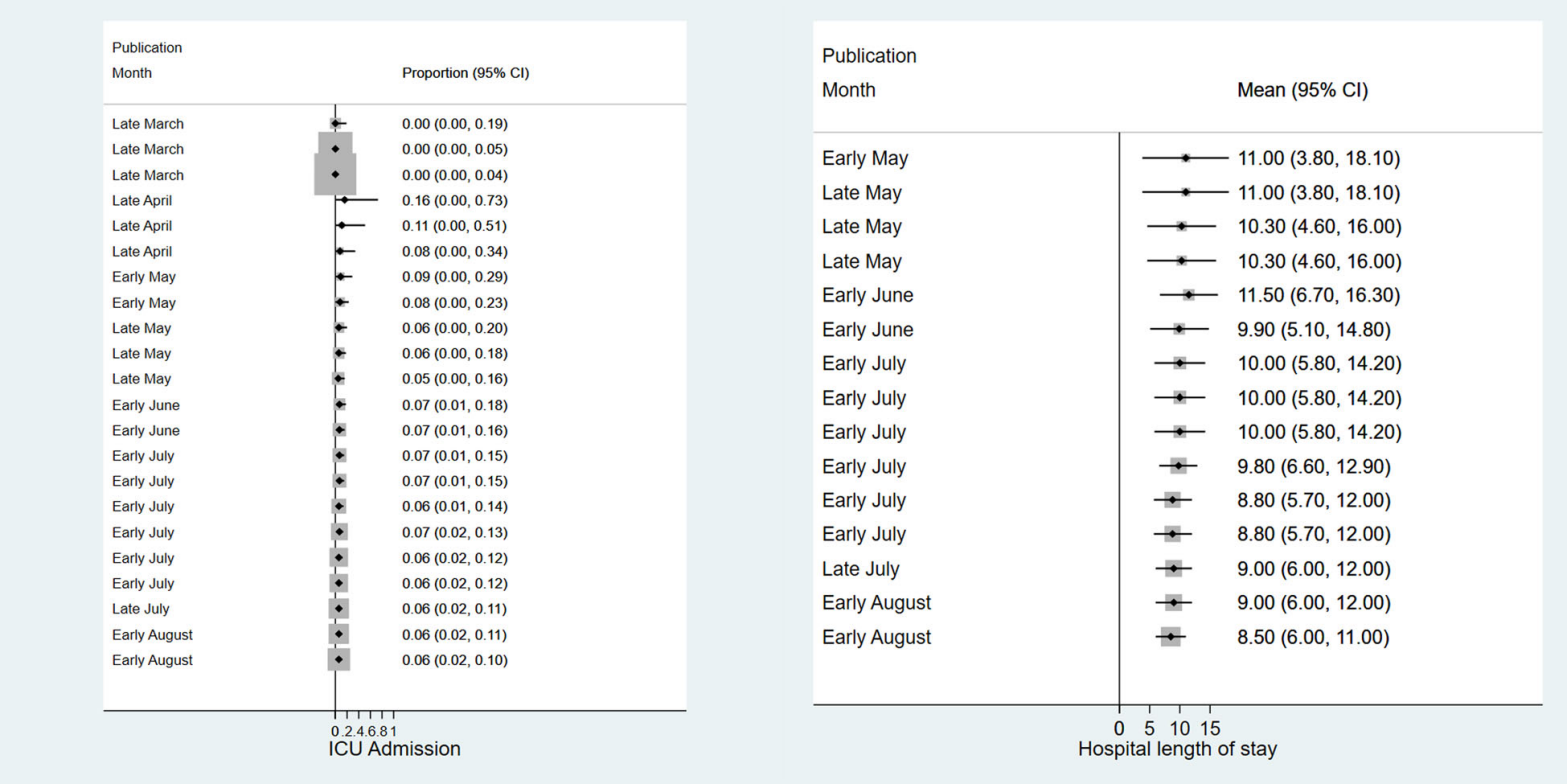
Pattern of intensive care unit admission and hospital length of stay of pregnant women infected with SARS-CoV-2 in case series studies

**Fig. 3:**
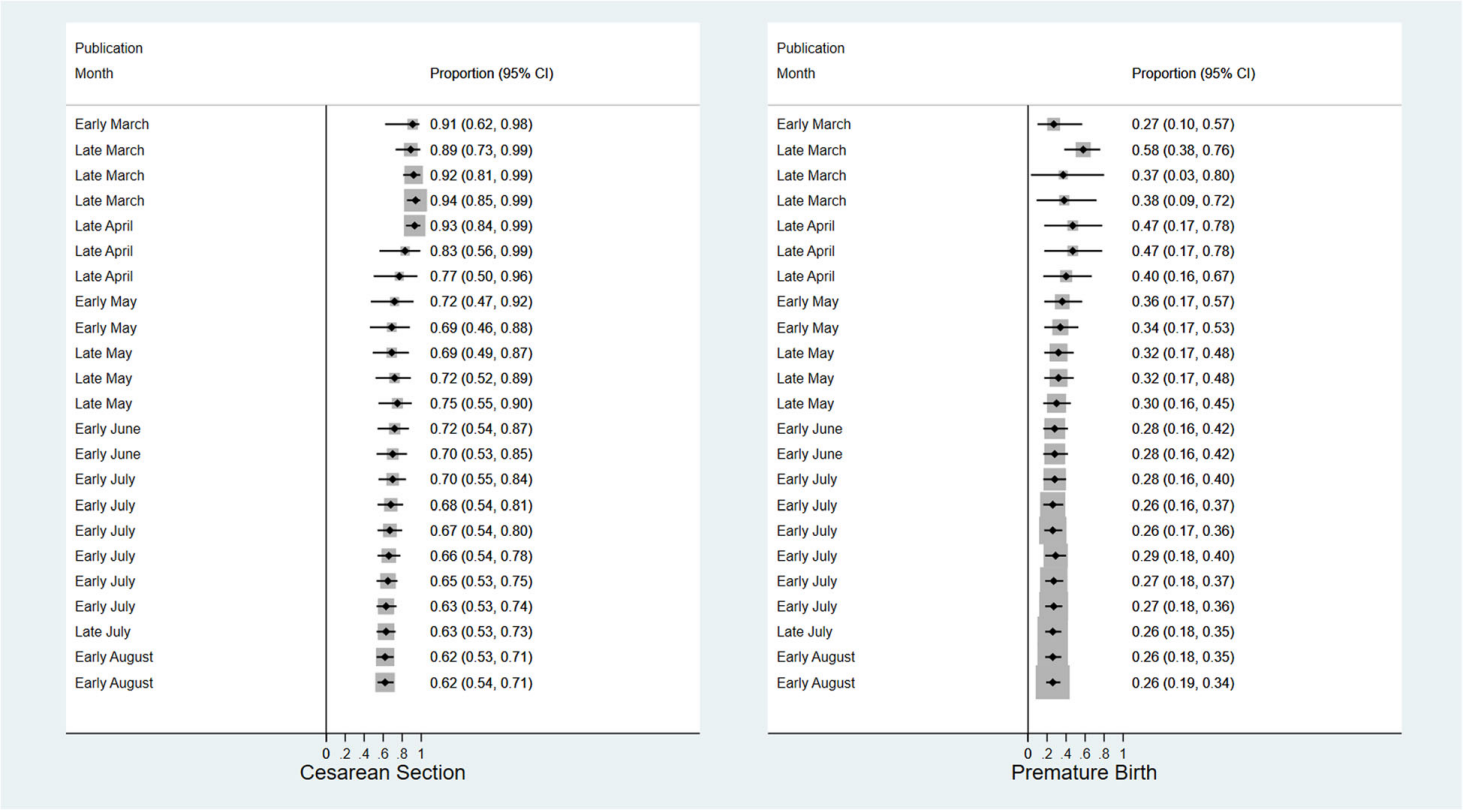
Pattern of cesarean section and preterm birth rates among pregnant women infected with SARS-CoV-2 in case series studies

**Fig. 4:**
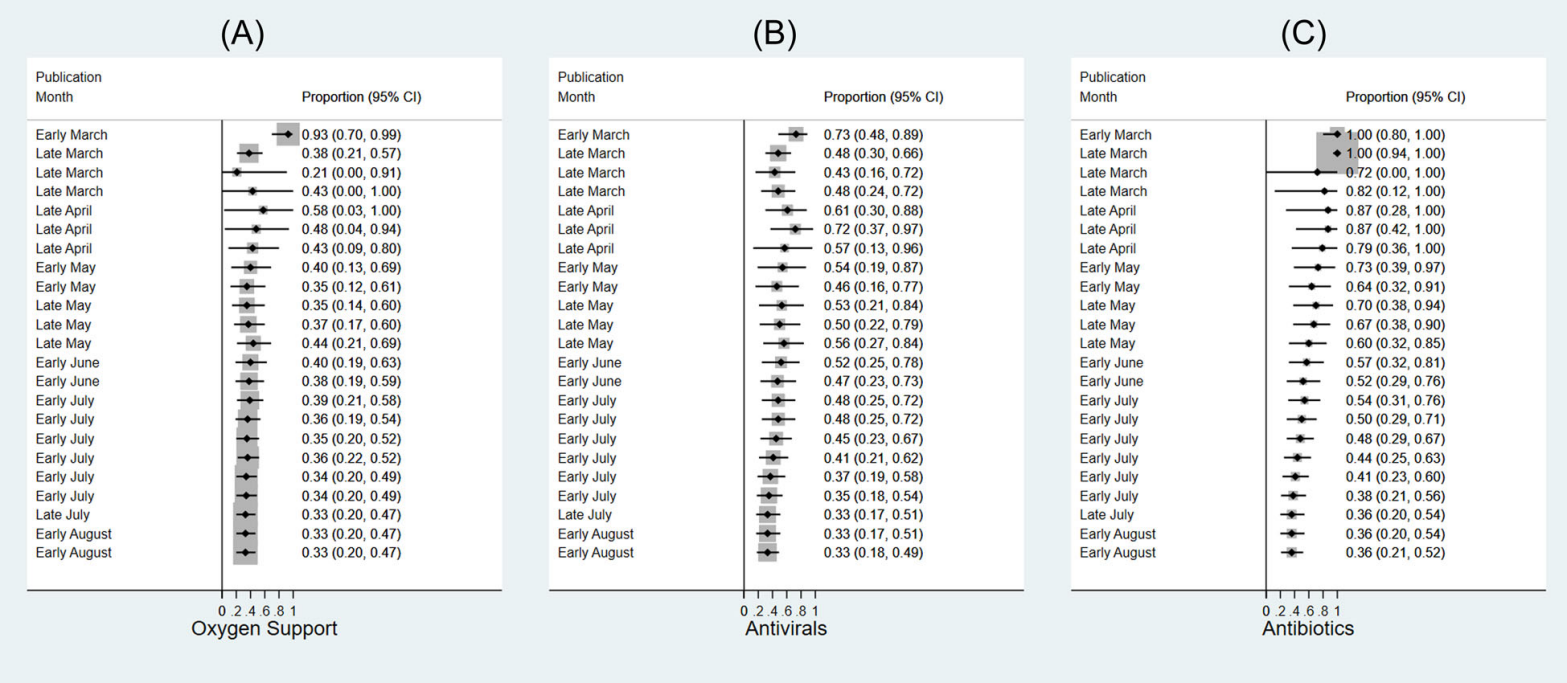
Pattern of oxygen support, antivirals, and antibiotics use among pregnant women infected with SARS-CoV-2 in case series studies

In general, a decline in the use of most therapeutics was observed over time except for HCQ and corticosteroid use. A slight increase in the use of HCQ and corticosteroids has started to begin since early May, 2020 (Supplementary Fig. 2). Like the decline in therapeutic use, the reduction in all outcomes (ICU admission, HLOS, and preterm birth and cesarean section rates) over time was also observed (Figs. 2 and 3). Among therapeutics, the greatest decline from May to August was 24% in antibiotics, 23% in antivirals, 11% in oxygen support (Fig. 4) while no change or slight gain in other therapeutics was observed. Only a 13% change in cesarean section and 2% change in preterm birth with 2 days reduction in HLOS were estimated between May to August without any difference in ICU admission and mechanical ventilation support.

### Subgroup analysis

The ICU admission rate, average HLOS, and preterm birth rate were found to be consistently increased in studies utilizing high proportions of immunosuppressants and antibiotics. Studies with high ICU admission and preterm birth rates were more likely to observe with high proportions of oxygen support and symptomatic patients. Studies with more ICU admitted subjects had also reported more usage of HCQ and anticoagulants. Increased ICU admission was also observed in studies with more obese patients. The preterm birth rate was higher in studies with more cesarean sections, more use of zinc/magnesium, and DM and HTN subjects while the preterm birth rate was less likely to observe in studies with low use of HCQ and no use of plasma therapy. The longest HLOS was noticed in studies using plasma therapy. Studies with fewer HTN patients had also reported decreased HLOS (Table 4).

**Table 4:** Meta-analysis of outcomes according to maternal and treatments characteristics

	Groups	ICU Admission	Hospital Length of Stay	Preterm Birth
		Proportion (95% CI)	Mean (95% CI)^a^	Proportion (95% CI)
**Treatment**				
Oxygen support	≤ 0.25 (low)	0.03 (0.00, 0.08)	8.89 (4.31, 13.48)	0.20 (0.10, 0.33)
	> 0.25 (high)	**0.10 (0.02, 0.21)**	9.10 (8.05, 10.15)	**0.33 (0.21, 0.46)**
Steroids	Not given	0.06 (0.01, 0.15)	7.45 (2.22, 12.69)	0.29 (0.17, 0.42)
	Given	0.06 (0.02, 0.11)	9.18 (6.49, 11.86)	0.27 (0.17, 0.37)
Immunosuppressants	Not given	0.03 (0.01, 0.06)	7.75 (5.10, 10.41)	0.26 (0.18, 0.36)
	Given	**0.24 (0.05, 0.51)**	**12.37 (4.22, 20.52)**	**0.35 (0.13, 0.60)**
Hydroxychloroquine	≤ 0.043 (low)	0.02 (0.00, 0.05)	9.95 (6.24, 13.65)	**0.30 (0.16, 0.46)**
	> 0.043 (high)	**0.11 (0.04, 0.21)**	7.30 (4.22, 10.38)	0.22 (0.15, 0.30)
Antivirals	≤ 0.25 (low)	0.05 (0.02, 0.09)	8.84 (5.63, 12.05)	0.29 (0.16, 0.44)
	> 0.25 (high)	0.07 (0.00, 0.20)	7.38 (4.67, 10.10)	0.25 (0.16, 0.34)
Zinc/Magnesium	Not given	0.06 (0.02, 0.11)	8.34 (5.36, 11.32)	0.26 (0.18, 0.34)
	Given	0.07 (0.03, 0.13)	NA	**0.44 (0.30, 0.58)**
Anticoagulants	Not given	0.03 (0.02, 0.05)	8.10 (4.98, 11.23)	0.27 (0.18, 0.38)
	Given	**0.18 (0.03, 0.40)**	9.24 (3.73, 14.74)	0.28 (0.14, 0.44)
Antibiotics	≤ 0.23 (low)	0.04 (0.01, 0.06)	6.37 (3.49, 9.24)	0.22 (0.13, 0.32)
	> 0.23 (high)	**0.09 (0.02, 0.20)**	**10.44 (6.23, 14.65)**	**0.31 (0.20, 0.44)**
Plasma/Anti-Liver damage	Not given	0.06 (0.02, 0.11)	7.74 (5.26, 10.22)	0.28 (0.20, 0.36)
	Given	0.04 (0.00, 0.22)	**13.99 (9.27, 18.70)**	**0.17 (0.05, 0.32)**
**Cesarean section**	≤ 0.51 (low)	0.06 (0.02, 0.12)	8.73 (5.11, 12.34)	0.21 (0.13, 0.30)
	> 0.51 (high)	0.06 (0.00, 0.15)	7.95 (5.45, 10.45)	**0.33 (0.20, 0.46)**
**Symptomatic**	≤ 0.94 (low)	0.04 (0.01, 0.08)	7.62 (3.80, 11.45)	0.18 (0.15, 0.22)
	> 0.94 (high)	**0.10 (0.02, 0.22)**	7.85 (5.17, 10.53)	**0.38 (0.23, 0.54)**
**Comorbidities**				
Diabetes mellitus	≤ 0.06 (low)	0.07 (0.02, 0.13)	8.32 (5.08, 11.55)	0.21 (0.14, 0.29)
	> 0.06 (high)	0.08 (0.00, 0.22)	8.62 (4.42, 12.82)	**0.39 (0.19, 0.61)**
Hypertension	≤ 0.03 (low)	**0.11 (0.01, 0.27)**	**10.81 (6.60, 15.01)**	0.27 (0.12, 0.44)
	> 0.03 (high)	0.04 (0.02, 0.07)	6.76 (4.54, 8.98)	**0.32 (0.18, 0.47)**
Asthma	≤ 0.034 (low)	0.07 (0.00, 0.22)	9.97 (5.63, 14.30)	0.35 (0.19, 0.53)
	> 0.034 (high)	0.07 (0.03, 0.13)	7.28 (3.95, 10.62)	0.24 (0.11, 0.40)
Obesity	≤ 0.10 (low)	0.04 (0.01, 0.08)	7.71 (3.58, 11.85)	**0.42 (0.10, 0.78)**
	> 0.10 (high)	**0.13 (0.03, 0.29)**	8.25 (4.37, 12.13)	0.33 (0.15, 0.53)

^*a*^*Weighted mean reported*; *ICU* intensive care unit, *CI* confidence interval, *NA* not available; bold values indicate a significant presence (at least 5%) of the condition relative to their average value

### Systematic review of case reports

The average age of patients was found to be similar among geographic locations while other maternal characteristics were found to be different across geographic locations (Table 5). The most common treatments in Asian studies were antibiotics (80%), antivirals (60%), oxygen support (40%), and steroids (40%) with the least usage of immunosuppressants (4%). In the US studies, the most frequent therapeutics were oxygen support (73%), steroids (71%), antibiotics (53%), antivirals (47%), and HCQ (40%). In European studies, oxygen support (69%), antibiotics (62%), steroids (46%), and zinc/magnesium (38%) were commonly used. HCQ (44%) and anticoagulants (22%) were frequently used in Latin American studies than in rest countries. The mechanical ventilation and ICU admission were most commonly observed in the US (67 and 80%) than European (38 and 38%), Latin American (33 and 33%), and Asian (16 and 28%) studies, respectively. However, the average HLOS was highest in Asian (19.1 days) and Latin American studies (14.2 days) while similar in the US and European studies (12.6 and 11 days respectively). The preterm birth rate was highest in the US (69%) followed by Europe (54%), Asia (44%) and Latin America (33%). The cesarean section rate was highest in Latin American studies (78%) but similar in other countries (68–69%). The other maternal outcomes were minimally observed in any country except the higher maternal death (33%) and fetal demise (56%) reported in Latin American studies. In association analysis (Supplementary Table 3), the use of antivirals and oxygen support was consistently associated with all adverse outcomes. In addition, HCQ and immunosuppressant treatments were associated with an increased proportion of ICU admission while the use of zinc/magnesium and steroids was associated with the increased preterm birth rate.

**Table 5:** Maternal characteristics, treatments, and outcomes among the 64 pregnant women by geographic locations

	Asia	USA	Europe	Latin America
	Proportion (95% CI)	Proportion (95% CI)	Proportion (95% CI)	Proportion (95% CI)
**Maternal Characteristics**				
Age (years); mean (95% CI)	30.00 (28.70, 31.20)	30.90 (27.30, 34.50)	32.8 (30.10, 35.40)	32.2 (29.50, 34.90)
BMI (kg/m^2^); mean (95% CI)	33.70 (5.20, 62.30)	37.0 (30.20, 43.90)	41.4 (32.70, 50.10)	28.9 (25.80, 32.00)
Gestational age (weeks); mean (95% CI)	36.20 (34.60, 37.80)	28.40 (25.50, 31.30)	33.4 (30.50, 36.30)	29.2 (25.80, 32.50)
**Symptomatic Presentation**	0.80 (0.59, 0.93)	1.00 (0.77, 1.00)	1.00 (0.75, 1.00)	0.78 (0.40, 0.97)
**Comorbidities**				
Diabetes mellitus	0.09 (0.00, 0.41)	0.40 (0.12, 0.74)	0.38 (0.14, 0.68)	0.00 (0.00, 0.34)
Hypertension	0.00 (0.00, 0.28)	0.10 (0.00, 0.45)	0.23 (0.05, 0.54)	0.00 (0.00, 0.34)
Asthma	0.00 (0.00, 0.28)	0.40 (0.12, 0.74)	0.00 (0.00, 0.25)	0.00 (0.00, 0.34)
Obesity	0.09 (0.00, 0.41)	0.56 (0.21, 0.86)	0.25 (0.05, 0.57)	NA
**Treatment Profiles**				
Oxygen support	0.40 (0.21, 0.61)	0.73 (0.45, 0.92)	0.69 (0.39, 0.91)	0.33 (0.07, 0.70)
Steroids	0.40 (0.21, 0.61)	0.71 (0.42, 0.92)	0.46 (0.19, 0.75)	0.11 (0.00, 0.48)
Immunosuppressants	0.04 (0.00, 0.21)	0.33 (0.12, 0.62)	0.00 (0.00, 0.25)	0.00 (0.00, 0.34)
Hydroxychloroquine	0.13 (0.03, 0.32)	0.40 (0.16, 0.68)	0.23 (0.05, 0.54)	0.44 (0.14, 0.79)
Antivirals	0.60 (0.39, 0.79)	0.47 (0.21, 0.73)	0.23 (0.05, 0.54)	0.33 (0.07, 0.70)
Zinc/Magnesium	0.12 (0.03, 0.31)	0.36 (0.13, 0.65)	0.38 (0.14, 0.68)	0.00 (0.00, 0.34)
Anticoagulants	0.08 (0.01, 0.26)	0.08 (0.00, 0.38)	0.15 (0.02, 0.45)	0.22 (0.03, 0.60)
Antibiotics	0.80 (0.59, 0.93)	0.53 (0.27, 0.79)	0.62 (0.32, 0.86)	0.44 (0.14, 0.79)
Plasma therapy/Anti-liver damage	0.20 (0.07, 0.41)	0.20 (0.04, 0.48)	0.08 (0.00, 0.36)	0.00 (0.00, 0.34)
Mechanical ventilation	0.16 (0.05, 0.36)	0.67 (0.38, 0.88)	0.38 (0.14, 0.68)	0.33 (0.07, 0.70)
**Maternal & Pregnancy Outcomes**				
ICU admission	0.28 (0.12, 0.49)	0.80 (0.52, 0.96)	0.38 (0.14, 0.68)	0.33 (0.07, 0.70)
HLOS (days) mean (95% CI)	19.10 (13.50, 24.80)	12.60 (9.50, 15.70)	11.00 (5.00, 17.00)	14.20 (2.30, 26.20)
Cesarean section	0.68 (0.46, 0.85)	0.67 (0.38, 0.88)	0.69 (0.39, 0.91)	0.78 (0.40, 0.97)
Maternal death	0.00 (0.00, 0.14)	0.00 (0.00, 0.22)	0.00 (0.00, 0.25)	0.33 (0.07, 0.70)
Fetal demise	0.00 (0.00, 0.14)	0.00 (0.00, 0.25)	0.00 (0.00, 0.25)	0.56 (0.21, 0.86)
Premature birth	0.44 (0.24, 0.65)	0.69 (0.41, 0.89)	0.54 (0.25, 0.81)	0.33 (0.07, 0.70)
Neonatal death	0.04 (0.00, 0.20)	0.00 (0.00, 0.21)	0.00 (0.00, 0.23)	0.00 (0.00, 0.34)

Note: Asia includes China, India, Iran, Jordon, and Korea; Europe includes France, Italy, Netherlands, Portugal, Spain, Sweden, Turkey, and United Kingdom; South America includes Brazil and Peru

*CI* confidence interval, *ICU* intensive care unit, *HLOS* hospital length of stay, *BMI* body mass index, *NA* not available, *USA* United States of America

## Discussion

In the meta-analysis of average-risk patients, the US studies revealed better maternal outcomes with more asymptomatic patients, more comorbidities, and less use of overall treatment interventions compared to the Asian and European countries. Furthermore, the average HLOS was also shorter in the US and European studies than in Asian studies without much increase in ICU admission or mechanical ventilation use. Over the course of the pandemic time-lapse, most therapeutic use for COVID-19 showed a declining pattern. The subgroup analysis showed that antibiotics and immunosuppressants were consistently associated with adverse outcomes in average-risk patients, while antivirals and oxygen support were associated with all adverse outcomes in severe cases. In addition, HCQ was also associated with more ICU admission in both average-risk and severe cases.

Our analysis of case reports and case series studies showed marked differences in clinical presentation and therapeutics. As case studies often report complex cases requiring critical management [19], our analysis of case reports also present therapeutics and outcomes of severe cases compared to case series analysis. Furthermore, the US and European studies presented more critically ill patients in their case reports compared to Asian and Latin American studies. The average age of pregnant patients was the early 30s in both case reports and case series analyses. Some recent reports identified multiple reasons for more COVID-19 detection in younger persons than older adults [88]. Recently a study showed that pregnant women with COVID-19 were more likely to be in the age of 25–34 years compared to non-pregnant women with COVID-19 [6]. As observed in our previous study [19], the US patients were more likely to be asymptomatic compared to the Asian and European patients. This could be due to more availability of testing of COVID-19 in pregnant women coming in for a routine follow up in the US. Breslin et al. [89] presented the importance of early screening for pregnant patients and their positive outcomes using a retrospective chart review. The ICU admission and preterm birth rates in our study were comparable to other studies [19, 90]. However, multiple studies [6, 91] have shown that pregnant women with COVID-19 were more likely to have hospitalization, ICU admission, mechanical ventilation use compared to non-pregnant women with COVID-19. Moreover, we also previously showed high rates of adverse pregnancy outcomes including preterm and cesarean section outcomes in pregnant women with COVID-19 [19] suggesting a scope of improvements in managing pregnant women.

We observed that antivirals and antibiotics were mostly used in Asian studies for managing all pregnant patients compared to other countries. Given the lack of efficacy data for most therapeutics of COVID-19 [92], the practice in the US and European countries might have been to minimally expose any therapeutics to average-risk pregnant patients. The highest use of antibiotics in Asian studies may be due to local guidelines for managing COVID-19 patients [93], suspicion of bacterial or fungal coinfections due to unavailability of rapid and affordable testing to differentiate viral and bacterial infections, and health-care-associated infections [94] due to prolonged hospitalization. In our analysis, a higher than anticipated use of antibiotics was also observed in the US and European studies. In a US study, more than half of the patients initially received antibiotics for the suspicion of bacterial infection, later in their stay, testing revealed that more than 96% of patients had the COVID-19 only and did not need antibiotics demonstrating their overuse [14]. Our study suggests that indiscriminate use of antibiotics needs to be minimized particularly in pregnant patients.

Our study showed a consistent pattern in the increased rates of ICU admission and preterm birth including longer HLOS associated with the increased use of antibiotics in average-risk patients. This observation could be due to health-care-associated infections requiring more antibiotics in ICU and hospitalized patients [94]. In addition, bacterial infections during pregnancy have been associated with an increased risk of preterm birth [95]. Although the association between the increased use of antibiotics in COVID-19 patients and antimicrobial resistance (AMR) is not clear [96], there may be likely to observe more AMR among COVID-19 patients. This may exacerbate the management of drug-resistant patients who are at-risk for bacterial infections. Though immunosuppressant was minimally used in the US and European studies, it was found to be associated with adverse clinical and maternal outcomes in our analysis. This finding was further supported by the analysis of case reports of the US studies. The use of immunosuppressants may yield significant complications and requires a proper assessment prior to its use [97]. Immunosuppressant use may be avoided in pregnant women unless it is clinically indicated.

The use of antivirals was associated with increased adverse outcomes in the analysis of case reports. Relatively lower use of antivirals in the US and European studies may be one of the reasons for having shorter HLOS compared to Asian studies. There have been studies where mixed effects of antivirals have been observed with potentially no benefits [98, 99]. As oxygen supplementation is required in the majority of critically ill patients [100], we also observed a high rate of ICU admission and preterm birth with a high proportion of oxygen support both in average-risk and severe cases of COVID-19. Although the reason for the potential association between oxygen therapy and preterm birth is unclear, the use of oxygen therapy was associated with an increased use of COVID-19 specific medications and inpatient mortality [101]. Given no evidence of optimal strategy for oxygen therapy in COVID-19 patients [99], a careful consideration is needed for oxygen therapy in pregnant patients. Similar to our study findings, HCQ has been associated with an increased risk of ICU admission [102] and the use of HCQ may be avoided in pregnant women from western countries. Consistent with our study, findings from multiple clinical trials indicated benefits from steroid use in COVID-19 cases and steroids have been recommended for treating severe and critical COVID-19 cases [11]. The anticoagulants were given rarely but mostly to ICU patients in both case series as well as case reports studies as it has been recommended for high-risk individuals only [15]. In our study, plasma therapy was found to be associated with longer HLOS which could be due to its emergency use authorization for hospitalized patients only. Patients who received zinc/magnesium had shorter HLOS in case reports but associated with increased preterm birth in all patients. The evidence is emerging for the potential benefits of zinc/magnesium in COVID-19 and it seems promising in cases who are at low risk of preterm birth [103, 104].

Like another study demonstrating a significant drop in most COVID-19 medications [101], our cumulative meta-analysis demonstrated considerable drop in most therapeutics and associated outcomes for COVID-19 in pregnant women. As health care professionals learned through their own experiences and evidence accumulated from clinical trials, the decline in the most common therapeutics associated with no clear evidence of benefits was observed. However, there is a scope for reduction in therapeutics for optimizing outcomes by risk stratification in pregnant patients. Our findings suggest that optimal outcomes may be achieved by avoiding unnecessary medications, minimizing therapeutics associated with adverse outcomes, and adopting other preventive measures.

### Strengths and limitations

Our systematic review and meta-analysis have some limitations. The main limitation is primarily the lack of high-quality data in the included studies with smaller sample sizes. However, our analysis included the largest series of studies in pregnant women with COVID-19. The estimates obtained from case reports studies may be biased despite having a fair number of case reports. Although we have used random effects models for estimating any characteristics and results reported according to geographic location, a substantial presence of heterogeneity in the estimates might introduce bias in the estimates. In the absence of non-pregnant women in included studies, our estimates for the periodical changes in treatment apply to pregnant patients only. Although the associations are mostly correlative not causative in this study, the observational evidence obtained from this study is useful for improving health care for pregnant patients. However, the results from subgroup and association analyses should be interpretated with cautions due to small sample sizes, complex interactions among treatments, and unadjusted analyses. Despite these limitations, our study is the first comprehensive study that provides the current therapeutic profile, their geographical distribution and chronological evaluation among pregnant women with COVID-19. We also associated the maternal and clinical outcomes with the current therapeutic use along with other maternal characteristics. Our study for the first time provides the geographical differences in maternal characteristics with therapeutics and pregnancy and clinical outcomes separately for average and severe cases of COVID-19.

## Conclusion

In summary, a considerable decline in preterm birth rate and average HLOS was observed over the pandemic period. The rates of ICU admission, preterm birth, and average HLOS were estimated to be relatively higher in pregnant women with COVID-19 worldwide and varied by geographic locations. Although a considerable decline in the use of antibiotics, antivirals, oxygen support, and immunosuppressants was noticed, minimizing the use of these therapeutics by risk stratification and careful consideration may further improve maternal and clinical outcomes. More evidence is required for the use of steroids, zinc/magnesium, and plasma therapy in pregnant women with COVID-19. Geographical differences in therapeutics with differential rates of maternal and clinical outcomes in both average-risk and severe COVID-19 cases were observed. Overall avoiding unnecessary treatments and early screening of asymptomatic pregnant women particularly in their 30s may minimize adverse consequences of COVID-19.

## Supplementary Information

**Additional file 1: Supplementary Table 1.** Methodological quality assessment for studies included in the meta-analysis. **Supplementary Table 2.** Assessment of small studies effects (publication bias). **Supplementary Table 3.** Associations of maternal characteristics and treatments with outcomes in case reports.

**Additional file 2: Supplementary Figure 1.** Funnel plots for proportions of intensive care unit admission and preterm birth outcomes.

**Additional file 3: Supplementary Figure 2.** Pattern of steroids and hydroxychloroquine use among pregnant women infected with SARS-CoV-2 in case series studies.

## Data Availability

The data that support the findings of this study are available with corresponding authors upon reasonable request.

## Abbreviations

HCQ: Hydroxychloroquine
ICU: Intensive care unit
HLOS: Hospital length of stay
D-L: Dersimonian and Laird
CI: Confidence interval
I^2^: Measure of heterogeneity
COVID-19: Coronavirus disease-19
PRISMA: Preferred reporting items for systematic reviews and meta-analyses
GLM: Generalized linear model
DM: Diabetes
HTN: Hypertension
AMR: Antimicrobial resistance
