# Supplementary material for: Current trends and geographical differences in therapeutic profile and outcomes of COVID-19 among pregnant women - a systematic review and meta-analysis
Source: BMC Pregnancy Childbirth. 2021 Mar 24;21:247. doi: 10.1186/s12884-021-03685-w (PMC7990381; doi:10.1186/s12884-021-03685-w)
Supplement: Supplementary file 1 — Additional file 1: Supplementary Table 1. Methodological quality assessment for studies included in the meta-analysis. Supplementary Table 2. Assessment of small studies effects (publication bias). Supplementary Table 3. Associations of maternal characteristics and treatments with outcomes in case reports. [file 12884_2021_3685_MOESM1_ESM.docx]

Supplementary Table 1. Methodological quality assessment for studies included in the meta-analysis

| **PMID** | **Author** | **Quality** |
| --- | --- | --- |
|  | **Case series** |  |
| 32151335 | Chen et al. | Good |
| 32186894 | Liu et al. | Good |
| 32249918 | Li et al. | Good |
| 32285380 | Liu et al. | Fair |
| 32360108 | Hantoushzadeh et al. | Good |
| 32428964 | London et al. | Good |
| 32696241 | Chen et al. | Good |
| 32433453 | Savasi et al. | Fair |
| 32438521 | Zeng et al. | Fair |
| 32439389 | Lokken et al. | Fair |
| 32632417 | San-Juan et al. | Fair |
| 32641013 | Zhang et al. | Good |
| 32553908 | Sentilhes et al. | Fair |
| 32553910 | Blitz et al. | Fair |
| 32633022 | Prabhu et al. | Fair |
| 32633712 | Vivanti et al. | Good |
| 32649784 | Gabriel et al. | Fair |
| 32682342 | Sahin et al. | Fair |
| 32689846 | Barbero et al. | Fair |
| 32701761 | Emeruwa et al. | Fair |
| 32743014 | Xu et al. | Fair |
| 32776309 | Oncel et al. | Fair |
| 32760169 | Nayak et al. | Fair |

Supplementary Table 2. Assessment of small studies effects (publication bias)

|  | **Publication Bias (Egger’s Test)** | | |
| --- | --- | --- | --- |
|  | **Coefficient** | **SE** | **P-value** |
| **Treatment** |  |  |  |
| Oxygen support | 1.92 | 1.04 | 0.082 |
| Steroid | -1.04 | 1.32 | 0.442 |
| Immunosuppressant | 0.09 | 0.26 | 0.729 |
| Hydroxychloroquine | -1.02 | 0.95 | 0.292 |
| Antiviral | 3.42 | 0.95 | 0.002 |
| Zinc/Magnesium | -1.18 | 1.38 | 0.405 |
| Mechanical ventilation | 0.36 | 0.46 | 0.45 |
| Anticoagulant | 0.37 | 0.54 | 0.506 |
| Antibiotic | 3.22 | 0.97 | 0.004 |
| Plasma therapy/Anti-liver damage | 0.93 | 0.60 | 0.136 |
| **Maternal & Pregnancy Outcomes** |  |  |  |
| ICU admission | 0.35 | 0.55 | 0.531 |
| Maternal death | 0.37 | 0.30 | 0.229 |
| Cesarean section | 0.66 | 0.47 | 0.174 |
| Fetal demise | 1.71 | 0.65 | 0.079 |
| Neonatal death | ID |  |  |
| Premature birth | 0.70 | 0.48 | 0.167 |
| Hospital stay | ID |  |  |
| **Comorbidities** |  |  |  |
| Diabetes mellitus | 0.32 | 0.19 | 0.114 |
| Hypertension | -0.35 | 0.31 | 0.277 |
| Asthma | -0.09 | 0.25 | 0.721 |
| Obesity | -0.83 | 0.53 | 0.143 |
| **Symptomatic** | 1.88 | 1.34 | 0.179 |

SE, standard error; ICU, intensive care unit; ID, insufficient data; a non-significant p-value indicates an absence of small sample size effect.

Supplementary Table 3. Associations of maternal characteristics and treatments with outcomes in case reports

|  | **Group** | **ICU Admission** | | | **Preterm Birth** | | | **Mean of HLOS(days)**  **(95% CI)** | **p-value** |
| --- | --- | --- | --- | --- | --- | --- | --- | --- | --- |
|  |  | **No; n (%)** | **Yes; n (%)** | **p-value** | **No; n (%)** | **Yes; n (%)** | **p-value** |  |  |
| **N** |  | **35** | **27** |  | **31** | **32** |  |  |  |
| **Maternal Characteristics** |  |  |  |  |  |  |  |  |  |
| Age (years), mean (SD) |  | 31.1 (3.5) | 31.2 (5.5) | 0.92 | 31.1 (3.2) | 30.7 (5.0) | 0.68 | -0.30* | 0.045 |
| BMI (kg/m^2^), mean (SD) |  | 33.7 (7.0) | 39.7 (8.9) | 0.11 | 32.2 (6.8) | 41.2 (6.9) | 0.010 | 0.28* | 0.34 |
| Gestation (week), mean (SD) |  | 34.0 (5.6) | 31.0 (5.1) | 0.035 | 33.3 (6.9) | 32.2 (3.9) | 0.44 | -0.017* | 0.91 |
| **Symptomatic presentation** | No  Yes | 7 (21%)  27 (79%) | 0 (0%)  27 (100%) | 0.012 | 7 (23%)  23 (77%) | 0 (0%)  30 (100%) | 0.005 | NA |  |
| **Comorbidities** |  |  |  |  |  |  |  |  |  |
| Diabetes mellitus | No  Yes | 15 (71)  6 (29) | 18 (82)  4 (18) | 0.42 | 16 (76)  5 (24) | 16 (76)  5 (24) | 1.00 | 15.8 (11.9, 19.7)  13.8 (7.2, 20.4) | 0.60 |
| Hypertension | No  Yes | 19 (90)  2 (10) | 20 (91)  2 (9) | 0.96 | 18 (86)  3 (14) | 20 (95)  1 (5) | 0.29 | 16.1 (12.7, 19.5)  9 (-2.9, 20.9) | 0.16 |
| Asthma | No  Yes | 19 (90)  2 (10) | 20 (91)  2 (9) | 0.96 | 18 (86)  3 (14) | 20 (95)  1 (5) | 0.29 | 15.9 (12.3, 19.4)  10.8 (2.2, 19.3) | 0.31 |
| Obesity | No  Yes | 15 (71)  6 (29) | 14 (70)  6 (30) | 0.92 | 16 (76)  5 (24) | 12 (63)  7 (37) | 0.37 | 14.9 (10.7, 19)  16 (8.5, 23.5) | 0.77 |
| **Treatment** |  |  |  |  |  |  |  |  |  |
| Oxygen support | No  Yes | 26 (74%)  9 (26%) | 3 (11%)  24 (89%) | <0.001 | 22 (71%)  9 (29%) | 7 (23%)  23 (77%) | <0.001 | 11.8 (7.7, 16.0)  16.5 (12.9, 20.1) | 0.088 |
| Steroids | No  Yes | 23 (66%)  12 (34%) | 11 (42%)  15 (58%) | 0.069 | 23 (74%)  8 (26%) | 11 (38%)  18 (62%) | 0.005 | 13.6 (10.2, 17.1)  15.0 (10.8, 19.1) | 0.63 |
| Immunosuppressants | No  Yes | 34 (97%)  1 (3%) | 21 (81%)  5 (19%) | 0.034 | 29 (97%)  1 (3%) | 25 (83%)  5 (17%) | 0.085 | 14.1 (11.1, 17.1)  15.7 (8.2, 23.2) | 0.69 |
| Hydroxychloroquine | No  Yes | 32 (91%)  3 (9%) | 13 (50%)  13 (50%) | <0.001 | 25 (83%)  5 (17%) | 19 (63%)  11 (37%) | 0.080 | 13.9 (10.3, 17.6)  15.0 (11.1, 19.0) | 0.69 |
| Antivirals | No  Yes | 23 (66%)  12 (34%) | 11 (41%)  16 (59%) | 0.050 | 22 (71%)  9 (29%) | 12 (40%)  18 (60%) | 0.015 | 11.6 (8.4, 14.9)  17.7 (13.6, 21.9) | 0.020 |
| Zinc/Magnesium | No  Yes | 28 (80%)  7 (20%) | 20 (77%)  6 (23%) | 0.77 | 28 (90%)  3 (10%) | 19 (66%)  10 (34%) | 0.020 | 15.3 (11.9, 18.6)  11.6 (8.0, 15.3) | 0.25 |
| Anticoagulants | No  Yes | 34 (97%)  1 (3%) | 18 (75%)  6 (25%) | 0.010 | 29 (94%)  2 (6%) | 22 (81%)  5 (19%) | 0.16 | 13.5 (10.5, 16.6)  23.0 (16.8, 29.2) | 0.011 |
| Antibiotics | No  Yes | 13 (37%)  22 (63%) | 9 (33%)  18 (67%) | 0.76 | 13 (42%)  18 (58%) | 9 (30%)  21 (70%) | 0.33 | 12.5 (7.8, 17.2)  15.9 (12.6, 19.3) | 0.22 |
| Plasma therapy/  Anti-liver damage | No  Yes | 31 (89%)  4 (11%) | 22 (81%)  5 (19%) | 0.43 | 28 (90%)  3 (10%) | 24 (80%)  6 (20%) | 0.26 | 13.5 (10.9, 16.1)  20.2 (10.3, 30.2) | 0.054 |
| Mechanical ventilation | No  Yes | 32 (91%)  3 (9%) | 8 (30%)  19 (70%) | <0.001 | 27 (87%)  4 (13%) | 12 (40%)  18 (60%) | <0.001 | 12.9 (9.5, 16.3)  16.8 (12.4, 21.2) | 0.14 |
| **Cesarean section** | No  Yes | 13 (37%)  22 (63%) | 6 (22%)  21 (78%) | 0.21 | 17 (55%)  14 (45%) | 2 (7%)  28 (93%) | <0.001 | 10.5 (5.0, 15.9)  16.2 (13.1, 19.3) | 0.060 |

HLOS, hospital length of stay; ICU: intensive care unit; CI: confidence interval; SD: standard deviation; NA: not available

Note: *, Pearson correlation coefficient
