# Supplementary figures and images for: Current trends and geographical differences in therapeutic profile and outcomes of COVID-19 among pregnant women - a systematic review and meta-analysis
Source: BMC Pregnancy Childbirth. 2021 Mar 24;21:247. doi: 10.1186/s12884-021-03685-w (PMC7990381; doi:10.1186/s12884-021-03685-w)

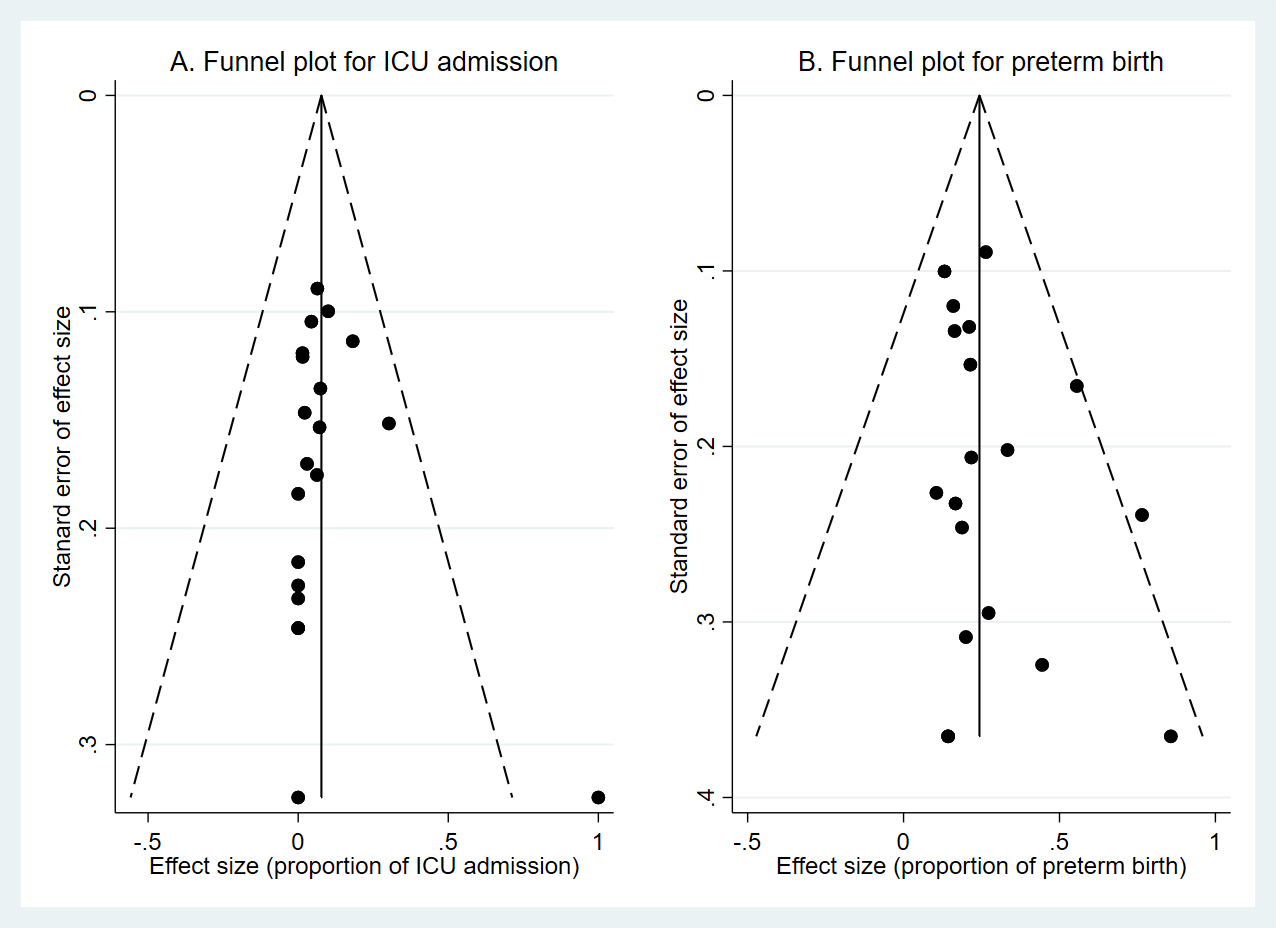

Supplement: Supplementary file 2 — Additional file 2: Supplementary Figure 1. Funnel plots for proportions of intensive care unit admission and preterm birth outcomes. [file 12884_2021_3685_MOESM2_ESM.tif]

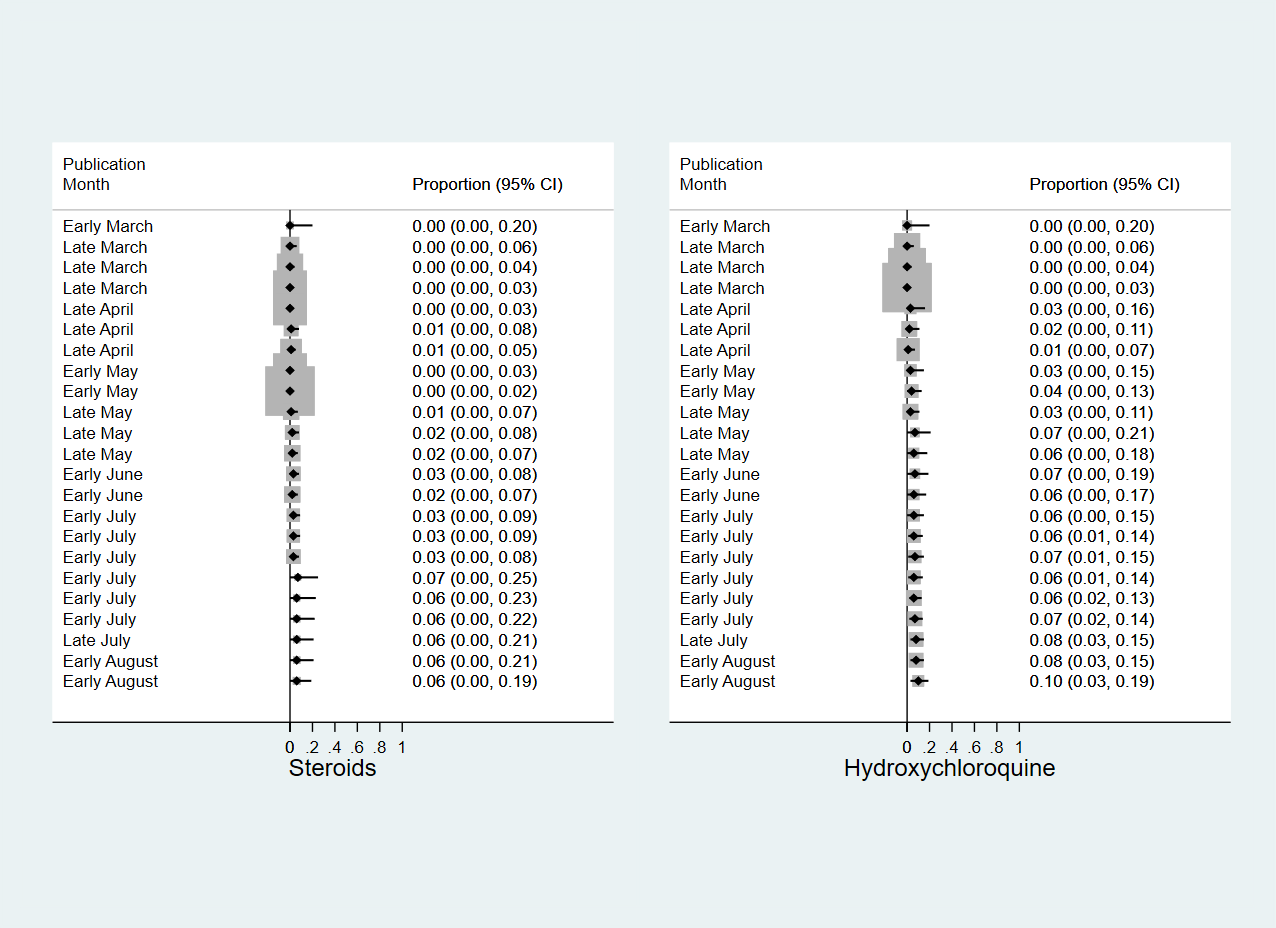

Supplement: Supplementary file 3 — Additional file 3: Supplementary Figure 2. Pattern of steroids and hydroxychloroquine use among pregnant women infected with SARS-CoV-2 in case series studies. [file 12884_2021_3685_MOESM3_ESM.tif]
